# Validation of neurodevelopmental assessments for early detection of high-risk infants in Zimbabwe: protocol for the Child Development Observation study (ChiDO study)

**DOI:** 10.1136/bmjpo-2025-004460

**Published:** 2026-09-19

**Authors:** Louisa Mudawarima, Nicole Santos, Gwendoline L Chimhini, Hilda A Mujuru, Rosemary Magonya, Rakesh Ghosh, Susanne P Martin-Herz

**Affiliations:** 1 Department of Child, Adolescent and Women’s Health, University of Zimbabwe Faculty of Medicine and Health Sciences, Harare, Harare Province, Zimbabwe; 2 Institute for Global Health Sciences, University of California San Francisco, San Francisco, California, USA; 3 Pediatrics, University of California San Francisco School of Medicine, San Francisco, California, USA

**Keywords:** Child Health, Children, Low and Middle Income Countries, Rehabilitation

## Abstract

**Introduction:**

Appropriate neurodevelopmental assessment tools are often unavailable in low- and middle-income countries (LMICs) due to lack of validation, cost and cultural-linguistic challenges. This reduces the availability of high-quality developmental evaluation, resulting in clinical gaps that impact individual child outcomes and modifiable risk detection for prevention. The objective of this study is to validate three neurodevelopmental assessment tools for early detection in low-resource settings through recruitment of infants across the neurodevelopmental impairment (NDI) risk spectrum related to perinatal asphyxia and neonatal encephalopathy (NE).

**Methods:**

This prospective, longitudinal cohort study in Harare, Zimbabwe will collect data at 3 months, 6 months, 12 months, 18 months and 24 months. The sample of approximately 600 caregiver-infant dyads will include: (1) infants without clinical concern for perinatal asphyxia or other complications (low risk); (2) infants who do not cry at birth and require resuscitation but do not have NE (moderate risk) and (3) infants who experience NE (high risk). In addition to demographic, antenatal and perinatal data collected at enrolment, neurodevelopmental assessments to be conducted include the Prechtl General Movement Assessment (GMA); Hammersmith Infant Neurological Examination (HINE); Global Scales for Early Development (GSED); Mullen Scales of Early Learning (MSEL) and caregiver-reported Ages and Stages Questionnaire. At the 24-month visit, a comprehensive diagnostic evaluation for NDI will be conducted adhering to international guidance. Predictive ability of all tools will be assessed by receiver operating characteristic (ROC) analyses, including area under the ROC curve. Construct validity of the GSED will be assessed by comparing to the MSEL. The GSED’s ability to differentiate subgroups defined by correlates of NDI risk will be assessed using generalised estimating equations.

**Ethics and dissemination:**

Ethical approvals have been obtained. If shown to be valid, the GMA and HINE could provide earlier, feasible and low-cost detection of children at elevated risk for NDI.

**WHAT IS ALREADY KNOWN ON THIS TOPIC**

- Paediatric neurodevelopmental assessment tools are often unavailable in low- and middle-income countries (LMICs), resulting in gaps that impact intervention access, clinical outcomes and availability of high-quality data for policy decisions.

**WHAT THIS STUDY ADDS**

- The objective of this study is to validate multiple tools for early detection of infants with neurodevelopmental impairment across the risk spectrum related to perinatal asphyxia and neonatal encephalopathy in Harare, Zimbabwe.

**HOW THIS STUDY MIGHT AFFECT RESEARCH, PRACTICE OR POLICY**

- If these tools are shown to be valid in this setting, they could provide earlier, feasible and low-cost detection of children at elevated risk for neurodevelopmental impairment (NDI) in LMICs.

## Introduction

 Developmental disorders are typically lifelong conditions with onset during childhood and adolescence, often associated with functional limitations. Early detection of neurodevelopmental impairment (NDI) is key to accessing early interventions at young ages, when brain plasticity and intervention effects are the greatest.^1–3^ Early detection is also important for identifying and reducing modifiable risk exposures for developmental disorders, thereby reducing poor outcomes, as well as for providing epidemiological data to inform policy decisions.^1 4^ Late detection of NDI means a critical window for intervention is missed for many children and their families.

More than 95% of the estimated 53 million children under 5 years of age with NDI worldwide live in low- and middle-income countries (LMICs), underscoring the crucial need for feasible and valid assessment tools in these contexts.^5 6^ However, it is increasingly recognised that available neurodevelopmental assessment tools are not adequately accurate, accessible and feasible for scaling in LMICs.^7 8^ High-quality developmental evaluation is hindered by complications of translation, cultural adaptation, cost and the lack of full, systematic evaluation of tool characteristics in these contexts. Additionally, vision, hearing and disability screening are notably missing in most assessments.^9–11^ The impacts of this knowledge gap extend across the individual, programmatic and population levels, likely leading to uncertain epidemiological estimates and poorer long-term outcomes for millions of children globally.^12 13^

Three neurodevelopmental assessment tools have shown promise but require longitudinal study in low-resource contexts. First, the Prechtl General Movement Assessment (GMA) has shown high sensitivity and specificity across several studies for subsequent cerebral palsy (CP) and predictive ability for cognitive delay when implemented under 5 months of age—though these studies have been in high-income contexts.^14^ GMA coding can be completed locally through on-site videos, caregiver-collected smartphone videos or during in-person evaluation, suggesting that it may be feasible to implement in remote contexts.^15^ Second, the Hammersmith Infant Neurological Examination (HINE) is administered between birth and 2 years of age and has also shown high sensitivity for CP (>90%) and for cognitive delay in at-risk infants.^16^ Both the GMA and HINE have been used in Asian and African LMICs and have the advantage of being observational examinations that are not language based, thus reducing the need for translation and adaptation in different contexts. However, very few studies have evaluated these tools’ ability to predict later neurodevelopmental outcomes in LMICs.^8 17^ Lastly, the Global Scales for Early Development (GSED) were created as developmental assessment instruments for population-level and programmatic evaluation of children ages 0–3 years.^18 19^ Designed to be globally applicable, easily adaptable, translatable and non-proprietary, GSED is in the final stages of validation with data collection in seven countries: Bangladesh, Brazil, Ivory Coast, the Netherlands, Pakistan, Tanzania and China.^20^ The GSED long form (LF) feasibility and initial validity for direct assessment has been explored in rural Western Kenya, with the GSED LF Developmental score (D-score) demonstrating concurrent validity among children 0–2 years with the Bayley Scales of Infant and Toddler Development, 3rd Edition and the Caregiver Reported Early Development Instruments.^21^

The objective of this study is to validate three neurodevelopmental assessment tools—the GMA, HINE and GSED—among a longitudinal cohort of children across the NDI risk spectrum related to perinatal asphyxia and neonatal encephalopathy in Harare, Zimbabwe. The study setting includes the Children’s Rehabilitation Unit (CRU), which opened in 1986 and provides children with developmental risks, delays or disorders and their families with free diagnostic and therapeutic services. Local data from the CRU Developmental Disability and Cerebral Palsy registers identify perinatal asphyxia as the most common attributed aetiology, leading to 33% of disability and 48% of CP.^22 23^ In the 10 years between 2006 and 2015, incidence by birth year (birth prevalence) of CP associated with perinatal asphyxia alone ranged from 1.0 to 2.3 per 1000 live births. However, despite a national at-risk referral system designed to identify infants at risk of NDI, CRU data between 2006 and 2015 also indicate a significant portion of children missed opportunities for early detection and intervention.^22 23^

Our specific aims are to:

1. Assess the ability of the GMA at 3 months and HINE at 3 months, 6 months, 12 months and 18 months to predict NDI at 24 months of age.
2. Assess the validity of the GSED and establish preliminary GSED threshold scores for predicting NDI at 24 months of age.

We hypothesise that both the GMA and HINE will be feasible to administer and show strong predictive ability for NDI at 24 months of age and that the GSED will show construct validity, discriminate between known risk groups and have predictive ability over multiple ages from 3–18 months for children with and without NDI at 24 months of age.

## Methods and analysis

### Study design

This is a prospective cohort study of infants across the risk spectrum for NDI related to perinatal asphyxia and neonatal encephalopathy. In addition to demographic, antenatal and perinatal data collected at enrolment, neurodevelopmental assessment will occur at 3 months, 6 months, 12 months, 18 months and 24 months of age.

### Setting and study population

The study will take place in Harare, Zimbabwe at eight of 12 municipal clinics, the neonatal unit (NNU) and CRU at Sally Mugabe Central Hospital. Recruitment of low-risk infants will be from city of Harare municipal clinics, medium-risk infants will be from municipal clinics, Sally Mugabe Central Hospital postnatal ward and the NNU and high-risk infants will be recruited from the NNU. Study visits will predominantly occur at municipal clinics to increase visit attendance, with final 24-month visits at the CRU to allow for vision examination and formal audiology evaluation.

### Study participants

A target sample of 696 infants will be recruited, with a final study sample size goal of n=600 completing study visits at 24 months of age. Inclusion and exclusion criteria by risk stratum are in table 1.

**Table 1:** Participant inclusion and exclusion criteria

Risk stratum	Target sample	Recruitment location	Description	Exclusion criteria
Low	250	Local clinics	No concern for perinatal asphyxia	Preterm birth (<37 completed weeks’ gestation) Low birth weight (<2500 grams) Confirmed sepsis, meningitis, severe jaundice (ie, infants with bilirubin level exceeding 425 IU (25 mg%) or symptoms of acute bilirubin encephalopathy) Significant congenital malformation, genetic syndrome Congenital infection by serology, culture or consistent clinical findings Residence outside urban/peri-urban Harare Primary caregiver <18 years
Moderate	223	Local clinics Sally Mugabe Central Hospital postnatal ward NNU Prior study cohort*	Did not cry at birth Required resuscitation Thompson score remained <6^37 38^	
High	223	NNU Prior study cohort*	Experienced perinatal asphyxia Admitted to the NNU with neonatal encephalopathy (ie, Thompson score 6 or more at any point after birth) Survived to hospital discharge without a potentially confounding illness	

* A parallel study focused on the Helping Babies Breathe/Essential Newborn Care intervention followed a cohort of high-risk infants until 6 months of age. A subset of these infants was approached for participation in the ChiDO study.

NNU, neonatal unit.

### Outcome measures

The primary outcome will be NDI at 24 months. Children meeting any one or more of the following criteria will be classified as having an NDI: (1) neurodevelopmental delay (Mullen Scales of Early Learning (MSEL) z-scores <−2 SD of mean), (2) disability on clinical examination by a certified neurodevelopmental paediatrician or a trained general paediatrician under supervision of a certified neurodevelopmental paediatrician using WHO International Classification of Diseases-11/10 (ICD-11/10) guidance and diagnostic criteria^24^ and Surveillance of Cerebral Palsy in Europe (SCPE) network guidelines for CP diagnosis^25^ or (3) sensory impairment assessed by formal audiological evaluation and standardised vision exam using Teller Acuity Cards (TAC).^26 27^ The MSEL was chosen as it has been successfully translated and used across several sub-Saharan African countries, including Zimbabwe and South Africa.^28 29^ Moreover, the study team has used this tool in another research study (manuscript under preparation), allowing us to leverage training and skilled human resources.

### Predictors

GMA, HINE and GSED scores will be evaluated as predictors of NDI.

### Sample size estimates

Each tool’s ability to predict NDI (aims 1 and 2) will be assessed using sensitivity, specificity and area under the receiver operating characteristic (AUROC) curve. Based on Ugandan data,^30^ 1.3% of low-risk and 29% of high-risk infants are expected to have NDI—approximately three and 59 children, respectively. For medium-risk infants, local unpublished data suggest a 5% NDI rate, or about 10 children, constituting an overall incidence of 12%. Assuming about 15% loss to follow-up, the two-sided 95% CIs for 0.85 sensitivity, specificity and AUROC estimable from this study will be 0.75 to 0.92, 0.82 to 0.88 and 0.79 to 0.91, respectively (table 2). Keeping other parameters unchanged, if higher loss to follow-up leads to a smaller analytic sample, the 95% CIs estimable from this study will be wider.

**Table 2:** Precision of point estimates for sensitivity, specificity and area under the ROC curve for n=600 (95% CIs)

Statistic	Estimate	CI	Estimate	CI	Estimate	CI
Sensitivity	0.75	0.64 to 0.86	0.85	0.76 to 0.93	0.95	0.87 to 0.99
Specificity	0.75	0.71 to 0.79	0.85	0.82 to 0.88	0.95	0.93 to 0.97
AUROC	0.75	0.68 to 0.82	0.85	0.79 to 0.91	0.95	0.92 to 0.99

AUROC, area under the receiver operating characteristic; CI, confidence interval.

### Patient and public involvement statement

Patients and/or the public were not involved in the design of this research.

### Data collection and assessment tools

Formal written informed consent will be obtained from caregivers following appropriate procedures. All consent documents will be available in both English and Shona (the local language) and consenting will take place in the caregiver’s preferred language. Only those who provide written consent will have their children recruited into the study. Participants who miss all 3-month, 6-month and 12-month visits will be administratively withdrawn from the study, since data at these time points are key to the study's main objectives.

A list of all study tools to be administered across enrolment and the five timepoint is in table 3.

**Table 3:** Overview of all proposed data collection tools by timepoint

Tool/assessment	Enrolment	3 months*	6 months	12 months	18 months	24 months
Eligible age window		>10 weeks, 1 day and <15 weeks, 6 days	6 months from DOB ± 14 days	12 months from DOB ± 14 days	18 months from DOB ± 28 days	24 months from DOB + 180 days
Enrolment form, risk stratum and randomisation assignment	X					
Pregnancy and delivery enrolment form	X					
Study visit form, including medical history, acute health status		X	X	X	X	X
Anthropometry		X	X	X	X	X
Ages and Stages Questionnaire		X	X	X	X	X
Mullen Scales for Early Learning		X	X	X	X	X
Prechtl General Movement Assessment		X				
Hammersmith Infant Neurological Examination		X	X	X	X	
Global Scales for Early Development (GSED) Long Form			X	X	X	X
GSED Short Form		X	X	X	X	X
Shona Symptom Questionnaire		X	X	X	X	X
Family care indicators		X		X		X
Washington Group/UNICEF Child Functioning Module						X
24-month diagnostic exam which includes visual and auditory screening						X

* The 3-month assessment is a hybrid model, with one in-person visit and one phone visit for caregiver-directed questionnaires.

DOB, date of birth.

At enrolment, we will collect the following data, if available:

- Maternal demographic and pregnancy information (eg, mother’s age and parity at delivery, family socioeconomic status, highest maternal education, number of antenatal care visits, maternal substance use history, maternal HIV status and illness during pregnancy such as hypertension or gestational diabetes).
- Perinatal history (eg, eclampsia, fever, chorioamnionitis).
- Labour characteristics (eg, duration of membrane rupture, labour and second stage, use of oxytocin/uterotonics, presentation at delivery, presence of shoulder dystocia, concern for foetal distress, meconium staining).
- Detailed delivery data (eg, mode of delivery, interventions, gestational age by last menstrual period and/or ultrasound, birth weight, neonatal outcome, Apgar scores if available).

At subsequent study visits, the following will be collected:

- Detailed medical and developmental histories since birth or last study visit (eg, feeding, growth, major illnesses, hospitalisations, immunisations, developmental milestones, any caregiver concerns).
- Support services received since birth or last study visit (eg, therapies or subspecialty medical care).
- Brief physical exam to include evaluation for newly identified dysmorphic features, chronic medical conditions or injury.
- Anthropometric measurements of length, weight, mid-upper arm circumference and occipital frontal head circumference completed using the GSED Anthropometry form.
- Neurodevelopmental assessments (see table 3).
- Shona Symptom Questionnaire (SSQ) to screen for maternal depressive symptoms.^29 31 32^
- Family Care Indicators (FCI) to collect information regarding the child’s home environment, such as stimulation by books, toys, etc.^33^

For each timepoint, children and their caregivers will have two visits at least 48 hours apart within their eligible age window (table 3). Children with acute illness at the time of their visit will be rescheduled within the allowable assessment window. The GSED LF and the MSEL will not be administered at the same visit or day, consistent with the GSED validation protocol.^20^ For example, on the first visit at each age, all history, physical examination and assessment tools except either the GSED Short Form (SF) and LF or ASQ and MSEL will be completed. At the second visit, the remaining assessment tools will be completed. Participants will be randomly assigned to a study arm which will dictate whether the GSED SF and LF or ASQ and MSEL will occur at the first visit, with half of the sample receiving GSED SF and LF first and half receiving ASQ and MSEL first. For randomisation, a list of study identification numbers will be randomly pre-assigned to study arms and stratified by risk level; as participants are consented, the local PI will assign participants to an arm based on their risk stratum. Caregiver-reported SSQ and FCI tools may be completed on either day, depending on length of visit and child/caregiver comfort, but the SSQ will only be completed at in-person visits. We estimate the first clinic visit will take approximately 1–1.5 hours, while the second clinic visit will be approximately 30–60 min. The phone call will take no more than 30 min.

At the final study visit (24 months), the caregiver-report Washington Group/UNICEF Child Functioning Module will be completed by interview.^34^ Additionally, a diagnostic evaluation for possible NDI adhering to WHO ICD-11/10 guidance^24^ and using SCPE network guidelines^25^ for CP diagnosis will be completed by a certified neurodevelopmental paediatrician or a trained general paediatrician with supervision of a certified neurodevelopmental paediatrician. This evaluation will include a comprehensive clinical history and physical examination including neurological and musculoskeletal systems, and vision screening using TAC.^26^ Hearing will be evaluated by otoacoustic emissions^27^ administered by an audiologist in the audiology department at the Harare Children’s Hospital at Sally Mugabe Central Hospital. Subspecialty evaluation and care for hearing and vision is freely available for children <5 years and referrals will be completed if needed based on this initial evaluation.

The study team will be trained in administration of the proposed tools, with additional refreshers and quality assurance activities offered at regular intervals. To assure fidelity of neurodevelopmental assessments, up to 5% of HINE, GSED and MSEL assessments at each age group conducted by each research nurse will be observed or video-recorded, dual scored and critiqued for quality control and assurance by tool-certified personnel. All GMA videos will be dual scored by two GMA advanced course trained and experienced coders, and scoring disagreements will be resolved by discussion and consensus. Study forms will be available in English and Shona (local language), following careful translation/backtranslation procedures. Translation is not necessary for the GMA and HINE, both of which do not include direct verbal questions. Prompts/questions administered to caregivers in Shona or English depending on caregiver preference. Prompts/questions for children will be in Shona.

### Data analysis

A score will be generated from each tool at each age of assessment. For example, a motor optimality score will be generated for each child using their GMA assessment data at 3 months, which will be analysed for predictive ability of the primary outcome at 24 months. A similar approach will be adopted with the other tools assessed at different ages. Assuming the composite primary outcome is the gold standard to measure NDI, sensitivity and specificity will be computed for each score at each age using different cut-offs. The age-specific approach will inform if any age(s) are better suited for administration of that tool to predict NDI at 24 months. Sensitivity versus 1-specificity will be plotted to generate ROC curves. To identify optimum threshold values for defining positive cases, various cut-offs of age-specific scores will be used to generate age-specific ROC curves and estimate the corresponding AUROCs which will be further explored to maximise statistical efficiency with clinical advantage.

Stata’s ROCREG procedure will be used, which allows for clustering of repeated measurements within participant and accounts for the influence of covariates. HINE and GSED’s performance will be assessed using the Stata ROCCOMP procedure, which specifically allows direct comparison of AUROCs in dependent samples at two different ages. Finally, we will compute AUROC for each instrument-age combination using the Stata ROCTAB procedure that computes sensitivity, specificity and percentage of cases correctly classified for each score on the predictive instrument.

Analysis to examine GSED’s predictive validity for NDI at 24 months will follow the same procedures as described earlier. Additionally, GSED construct validity at each age will be assessed by comparing it to the MSEL using Pearson correlation coefficient and mixed effects linear regression models. Covariates to be included in the adjusted models are prespecified in the study protocol and include maternal education, caregiver mental health (measured by the SSQ), birth weight, HIV exposure status, postnatal onset of serious health conditions and child home environment (measured by the FCI). These models will also allow exploration of potential interactions between GSED scores and the prespecified covariates.

To check consistency between dual scored items/scores, we will estimate various reliability measures. For continuous total or domain scores (eg, HINE, MSEL), intraclass correlation coefficients will be estimated. For categorical outcomes, Cohen’s kappa (or weighted kappa for ordinal categories) and percentage agreement will be quantified.

#### Missing data

All ROC analyses will include the participants that complete the diagnostic exam at 24 months. However, sensitivity analyses will be conducted where attrition-unrelated missing data will be replaced on a per analysis basis using multiple imputation algorithms. The proportion of missing data will be examined overall, by variable, by time point, and by treatment arm (if applicable). We will examine patterns of missingness through descriptive statistics and visual inspection, including the percentages and distribution of missing values and whether missingness is related to observed variables (eg, participant characteristics, previous assessments, or study site). These descriptions will be reviewed prior to any imputation. Based on the observed patterns, we will assess whether data are likely missing completely at random, missing at random (MAR) or missing not at random. Where missing data are judged to be attrition-unrelated and the MAR assumption appears plausible, multiple imputation by chained equations will be performed on a per-analysis basis. This approach makes the relatively mild assumption that missingness is related to observed data, an assumption often reasonable in longitudinal studies because the best predictor of a later outcome is typically its own prior measurement(s).^35^ To make the MAR assumption more plausible, auxiliary variables predictive of the variables with missing data and missingness itself will be included in the imputation model. Research shows multiple imputation assuming MAR performs well for ROC curve analyses, particularly for sample sizes >100.^36^

#### Data management

Data will be entered directly into a University of Zimbabwe (UZ) secure REDCap database and a UZ secure ODK server for GSED-related data or collected on paper forms and entered promptly by research staff. Only authorised research team members from University of California San Francisco (UCSF) and UZ will have access to both REDCap and ODK data. Digital videos for GMA assessment or QA videos will be labelled only by study ID number, visit age and date of collection. They will be stored on a secure cloud-based server accessible only to necessary research personnel. All study computers/tablets will be encrypted and password protected. No personal devices will be used with PHI. Paper records of data collection forms will be kept in double locked filing cabinets in locked office suites. Signed informed consent forms will be filed separately in a secure, locked location.

## Ethics and dissemination

Ethical approvals have been received from the UCSF Institutional Review Board (#21-34337), Sally Mugabe Central Hospital, Medical Research Council of Zimbabwe (MRCZ/A/2825), City of Harare, Research Council of Zimbabwe (RCZ Registration No. 05077) and the WHO Research Ethics Review Committee. We do not anticipate risks during this study, though it is possible caregivers may feel stress/concern if their child is not developing or growing well, or if they feel uncomfortable talking about their mental health, home environment, etc. Additionally, multiple study visits and the cumulative time for assessment may cause stress or time lost from work; we have included participant travel support and small refreshments at each visit.

### Referrals

If any concerns arise during data collection procedures, study staff will notify the local Principal Investigator, a neurodevelopmental paediatrician. Families will be informed of any clinical diagnoses or NDI. If medical conditions are detected in the infant, they will be referred to the local paediatric clinics. If developmental delays are identified, families will be referred to CRU services, such as occupational therapy, physiotherapy, speech therapy and family/caregiver support and counselling. CRU services are available at no cost to families.

### Significance and relevance

If shown to be valid, the GMA and HINE could be implemented in LMICs and provide much earlier, feasible and low-cost detection of children at elevated risk for NDI. They have the potential to markedly reduce the age at which children with or at risk for NDI are identified, allowing appropriate early intervention to be initiated by 6 months of age or earlier. Their potential for use by multiple levels of healthcare workers in LMICs can help to ensure that early detection occurs at both urban and rural health centres, promoting equity in access. Study findings will also contribute to international GSED validation efforts, including those for individual-level use of GSED, supporting improved neurodevelopmental assessment on a global scale. Specific to Zimbabwe, validation of these tools, along with use of the Washington Group/UNICEF Module on Child Functioning, will also support the goal of obtaining high quality data on NDI. Finally, validation in the proposed sample will set the stage for rapid evaluation, clinical use and research implementation with other at-risk groups (eg, in preterm infants, severe hyperbilirubinaemia or acute malnutrition).

## Conclusion

In Shona, the local language of Harare, Zimbabwe, chido translates to ‘something loved’. This study, the Child Development Observation Study—the ChiDO Study—aims to inform more widespread, feasible neurodevelopmental assessment in LMICs to ensure that every child and family has an opportunity to thrive and receive the resources they need.
