# Supplementary material for: Validation of neurodevelopmental assessments for early detection of high-risk infants in Zimbabwe: protocol for the Child Development Observation study (ChiDO study)
Source: BMJ Paediatr Open. 2026 Sep 19;10(1):e004460. doi: 10.1136/bmjpo-2025-004460 (PMC13599960; doi:10.1136/bmjpo-2025-004460)
Supplement: Supplementary file 1 [file bmjpo-2025-004460.reviewer_comments.pdf]

PEER REVIEW HISTORY

BMJ Paediatrics Open publishes all reviews undertaken for accepted manuscripts. Reviewers are asked to complete a checklist review form and are provided with free text boxes to elaborate on their assessment. These free text comments are reproduced below.

ARTICLE DETAILS

**TITLE (PROVISIONAL)**	Validation of neurodevelopmental assessments for early detection of high-risk infants in Zimbabwe: protocol for the Child Development Observation Study (ChiDO Study)
**AUTHORS**	Mudawarima, Louisa<br>Santos, Nicole<br>Chimhini, Gwendoline Lilly<br>Mujuru, Hilda A.<br>Magonya, Rosemary<br>Ghosh, Rakesh<br>Martin-Herz, Susanne

VERSION 1 – REVIEW

**REVIEWER NAME**	Tann, Cally
**REVIEWER AFFILIATION**	
**REVIEWER CONFLICT OF INTEREST**	No Conflict of Interest
**AI DISCLOSURE**	No, I have not used AI
**DATE REVIEW RETURNED**	22-Jan-2026

**GENERAL COMMENTS**	accept

VERSION 1 – AUTHOR RESPONSE

__**Response to reviewer**__
1. The primary outcome in this study is described as a composite of developmental delay on MSEL, clinician diagnosed disability, hearing impairment or vision impairment. I wonder if the authors might want to state explicitly "Children meeting any one of the following criteria will be classified as having an NDI". It is implied but not so explicit.
Response: Thank you for this suggestion. We have edited this text on page 7 (Outcome measures) per the reviewer's comment to indicate that any one or more of the criteria being met would lead to a classification of NDI.

2. The protocol does mention dual scoring of GMA, observation of HINE, GSED, MSEL. They might want to explain how this will be quantified (if they can) e.g. inter-rater agreement will be evaluated using x… as appropriate.
Response: Thank you for this suggestion. We have included additional detail on how dual scoring and inter-rater reliability will be evaluated. At the end of the data analysis section (page 11), we have included detailed quantification of how dual scoring and inter-rater reliability will be evaluated.

3. In terms of the missing data section, the authors might want to mention that they might look at the proportions and patterns of missing data and describe these prior to any imputation.

Response: We have expanded the missing data section to explicitly state that we will first examine the proportion and patterns of missingness prior to deciding on any imputation strategy. We have included additional information on page 12 of the manuscript.

4. It looks like the co-variates are pre-specified. Do the authors want to make this clearer by stating this?
Response: We have clarified that the covariates are pre-specified in the protocol. Additional text addressing this point is included on page 11 of the manuscript.

5. The authors may want to mention why MSEL has been chosen as the construct validity comparator, e.g. it was selected as it is in an established, comprehensive developmental assessment with xx psychometric validation… or something to make clearer why chosen as the comparator.
Response: In the paragraph related to Outcome measures (page 7), we have included our rationale for selecting the MSEL as part of the primary outcome composite. It has been used extensively in the sub-Saharan African region (see new references #30-31). Moreover, our research team has used the MSEL in a separate research study with great success, allowing us to leverage learnings and training infrastructure.

6. A few small consistencies e.g. NDV vs NDI – might just check and low-resource vs low resource
Response: Thank you for this suggestion. We have opted to write out NDV (neurodevelopment(al)) in the text and keep NDI for clarity. We have also checked for additional copy edit/typo inconsistencies.

7. A few references need checking (e.g., Mudawarima L PGCGNGSNMHS... Powell G NGLKBKMHS... Lanzoni M MSDMEVKOAAC).
Response: Thank you for catching these errors. We have made these corrections, as well as re-numbered citations given the addition of two new references (see #5 above).
